# Assessment of global antimicrobial resistance campaigns conducted to improve public awareness and antimicrobial use behaviours: a rapid systematic review

**DOI:** 10.1186/s12889-024-17766-w

**Published:** 2024-02-06

**Authors:** Ellie L. Gilham, Nicola Pearce-Smith, Vanessa Carter, Diane Ashiru-Oredope

**Affiliations:** 1 HCAI and AMR Division, Health Security Agency, London, UK; 2 The AMR Narrative, Altrincham, UK; 3 School of Pharmacy, University of Nottingham, Nottingham, UK

**Keywords:** Public campaign, Antibiotic resistance, AMR, Awareness, Health knowledge and attitudes, Behaviour change

## Abstract

**Introduction:**

Public health campaigns with a well-defined outcome behaviour have been shown to successfully alter behaviour. However, the complex nature of antimicrobial resistance (AMR) creates challenges when evaluating campaigns aimed at raising awareness and changing behaviour.

**Aims:**

To determine what campaigns have been conducted and which reported being effective at improving awareness of antimicrobial resistance and changing behaviour around antimicrobial use in members of the public. It also sought to determine the outcome measures studies have used to assess campaign effectiveness.

**Methods:**

A systematic search of Ovid MEDLINE and Embase, was conducted in October 2022 using a predefined search strategy. Studies which were published between 2010 and September 2022 that outlined a campaign or invention aimed at the public and focusing on AMR or antibiotic usage were eligible for inclusion and studies which solely targeted healthcare professionals (HCP) were excluded.

**Results:**

Literature searches retrieved 6961 results. De-duplication and screening removed 6925 articles, five articles from grey literature and reference screening were included, giving a total of 41 studies and 30 unique interventions. There was a distribution of campaigns globally with the majority run in Europe (*n* = 15) with most campaigns were conducted nationally (*n* = 14). Campaigns tended to focus on adult members of the public (*n* = 14) or targeted resources towards both the public and HCPs (*n* = 13) and predominately assessed changes in knowledge of and/or attitudes towards AMR (*n* = 16). Campaigns where an improvement was seen in their primary outcome measure tended to use mass media to disseminate information, targeted messaging towards a specific infection, and including the use of HCP-patient interactions.

**Discussion:**

This review provides some evidence that campaigns can significantly improve outcome measures relating to AMR and antibiotic usage. Despite a lack of homogeneity between studies some common themes emerged between campaigns reported as being effective. However, the frequent use of observational study designs makes it difficult to establish causation between the campaign and changes seen in the studies outcome measures. It is important that clear evaluation processes are embedded as part of the design process for future campaigns; a campaign evaluation framework for use by campaign developers may facilitate this.

**Supplementary Information:**

The online version contains supplementary material available at 10.1186/s12889-024-17766-w.

## Introduction

Antimicrobial resistance (AMR) has become a major public and global health challenge with an estimated 4.95 million deaths associated with bacterial AMR in 2019 [1]. Following the publication of a Global Action Plan in 2015 and subsequent UN declaration signed by 193 countries, several countries have published and are implementing national policies to tackle AMR [2, 3]. 

Inappropriate prescribing, especially for viral infections, such as respiratory tract infections (RTIs) [4], is one of the main driving factors for increasing levels of AMR. Most antibiotics are prescribed in primary care [5], a setting with significant interaction between the healthcare professional (HCP) and patient. Patient expectation and perceived patient expectation are often cited by general practitioners (GPs) as the main reasons for prescribing an antibiotic to treat a RTI [6, 7]. A meta-analysis showed that physician’s perception of patient desire for antibiotics and patient desire for antibiotics were associated with antibiotic prescribing for RTIs (odds ratio (OR): 2·11 to 23·3 and 0·61 to 9·87 respectively) [6]. In addition to the potential influence patients have during HCP-patient interactions in primary care, public behaviours such as self-medication [8] and inappropriate disposal of antibiotics [9] can also impact on AMR. Therefore, the lack of public knowledge regarding AMR and appropriate antibiotic usage, which has been identified through public surveys, is concerning [10]. 

Public health campaigns have been successful in improving knowledge and changing behaviour in other areas of public health [11, 12]. However, developing a public health campaign focused on AMR is challenging due to the complexity of both campaign messaging and the behaviour contributing towards increasing AMR. This also presents challenges when evaluating the campaign as there are certain scenarios where antibiotic prescribing is necessary and appropriate and some outcome measures may not reflect individual behaviours. Furthermore, previous reviews have restricted campaigns included by study design of the evaluating study [13], type of outcome measure used [14], or type of intervention implemented [3], and therefore do not provide an overall summary of campaigns and interventions which have been conducted to alter behaviour around antibiotic usage.

Therefore, this review aimed to determine what public campaigns to improve public awareness and antimicrobial use behaviours have been conducted worldwide and which of these have been effective at improving awareness of AMR or changing behaviour around antimicrobial use in members of the public. It also sought to determine what outcome measures were used to assess whether the campaign effectively altered factors known to contribute towards AMR.

## Methods

The review follows the PRISMA 2020 item checklist (Supplementary Material 1.) and is registered with PROSPERO (registration number CRD42022371142). The rapid review methodology was chosen due to the need to disseminate findings quickly to inform the development of the future AMR National Action Plan, however, this methodology does come with some limitations [15]. Abbreviated methods were followed with papers screened by one reviewer and 4% of full text screened papers reviewed by a second reviewer. Data extraction was also conducted by one reviewer. Given the heterogeneity of study designs and outcome measures, data was synthesised using a narrative synthesis.

### Search strategy

A PICO framework [16] was used to develop the main search terms, search strategies are shown in Supplementary Material 2. The database searches were performed on 5 October 2022 using Ovid Medline ALL and Ovid Embase with date limits 1 January 2010 to 4 October 2022. Conference abstracts were removed from the Embase search before download.

All records were imported into an Endnote library; after deduplication there were 4755 unique records. All 4755 citations were then imported into Rayyan, and 50 further duplicates were removed. Additionally, a grey literature search of Google identified 73 further unique citations and the reference lists of four systematic reviews identified from the search [3, 13, 14, 17] were screened to identify nine additional papers for screening. These citations were screened on title and abstract, and 557 (475 from published literature, 82 from grey literature and citation searching) were included for full text analysis. A PRISMA diagram (Supplementary Material 3.) depicts the flow of information through the review.

### Screening

Due to the large number of articles retrieved by the search, articles were initially screened by searching for the terms campaign and/or intervention in Rayyan with articles included for further consideration if they included these terms. Following this, abstracts were reviewed by one reviewer to determine whether the paper met the inclusion criteria. If insufficient information was included in the abstract to determine whether the paper met the inclusion criteria the full paper was screened. For any papers where it was not clear if they fully met the inclusion criteria consensus was gained from a second reviewer.

### Selection criteria

Studies which outlined a campaign or invention aimed at the public or patients and focusing on AMR or antimicrobial usage were considered. All study designs (except systematic reviews) and outcome measures were included, to allow data to be gathered on methods to assess measurable behaviour change resulting from an AMR campaign or intervention. Studies which solely targeted HCP were excluded to distinguish between interventions aimed at HCP and at the public. The inclusion and exclusion criteria applied can be found in Table 1.

**Table 1:** showing the study inclusion and exclusion criteria

	Inclusion	Exclusion
Language	English	Non-English
Time period	January 2010 to September 2022	Pre-2010
Intervention	Campaign or intervention focused on conveying information about AMR, antibiotic resistance and/or antibiotic usage	Campaigns or interventions focused on other topic areas
Population	General public and/or patients	Healthcare professionals only

### Data extraction

A data extraction tool was designed with specified column headings using Microsoft Excel. Data was extracted in line with the main aims of the review to provide information on the study design, country within which the campaign was conducted, the study sample, intervention (main components, year of implementation, duration and key messages), the intervention target population (public, patient or combined), campaign or intervention type (international, national, community or local), the comparator used to determine effectiveness, outcome measures used (antibiotic prescribing or consumption, knowledge, attitudes and awareness of AMR, behaviour change and campaign recognition), and the study’s key findings.

## Results

The literature search produced 6961 results. De-duplication removed 2206 results and the titles of the remaining 4705 results were screened resulting in the removal of a further 4230 studies. Following this, 475 results underwent further screening, with 36 studies meeting the inclusion criteria. An additional nine results were identified through systematic review bibliography screening, and 73 during grey literature searches, of which five results met the inclusion criteria, giving a total of 41 studies (30 unique interventions) to be included (Supplementary Material 3.).

**Fig. 1:**
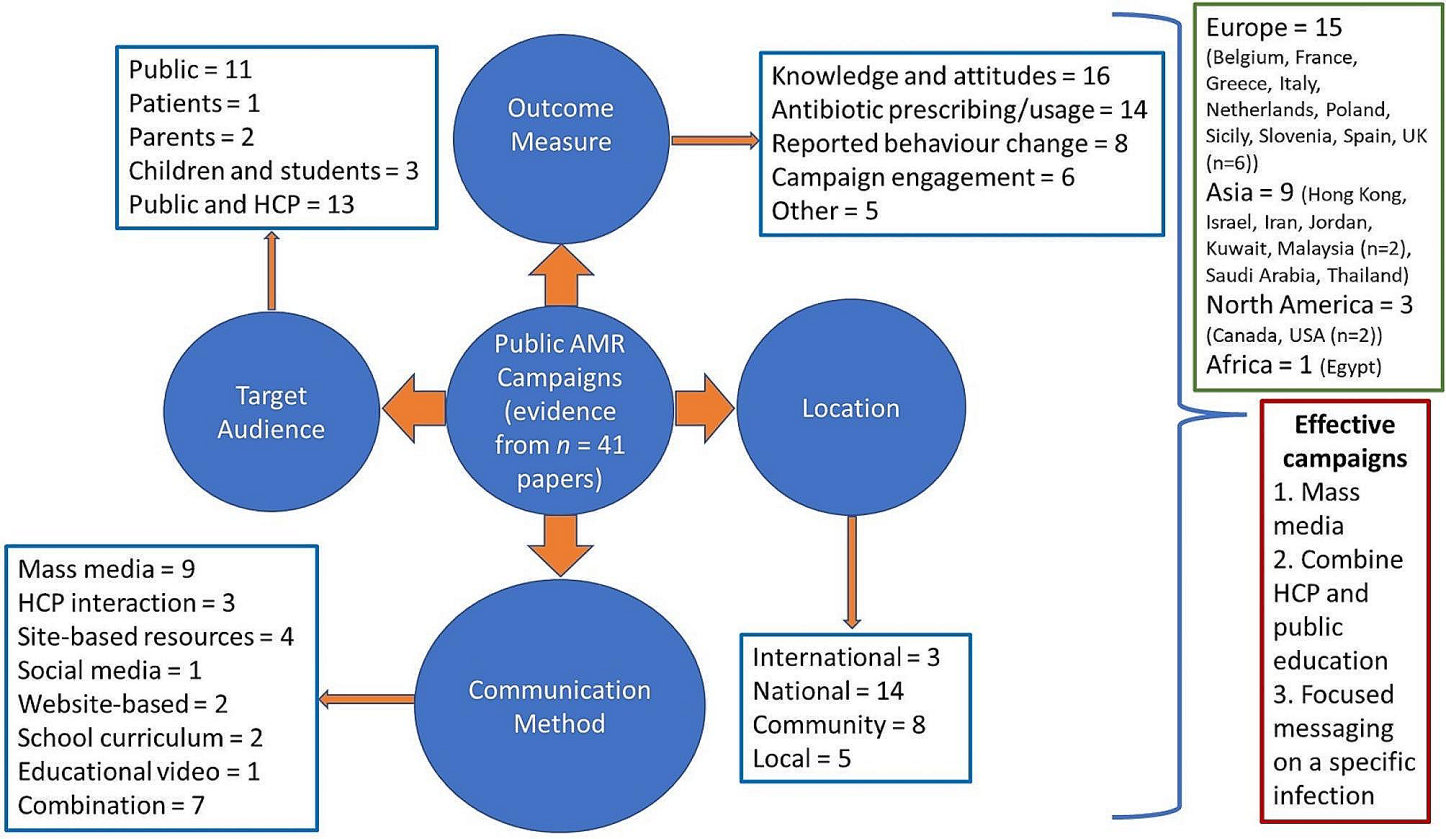
Characteristics of campaigns included within the review

Table 2. provides an overall summary of the studies included within this review and Table 3. Outlines the key focus of the included campaigns. The search showed a range of countries, both high and middle income, within which AMR campaigns or interventions had been conducted. However, most campaigns were conducted in high income countries (*n* = 23) [18–40], with six campaigns conducted or originally developed within the UK [35, 39–43], and four [44–47] and two [48, 49] campaigns conducted in upper and lower middle-income countries respectively. A summary of the campaign characteristics is available in Fig. 1.

**Table 2:** showing a summary of included campaigns and interventions

Intervention/ Campaign	Authors	Country	Campaign Year and Duration	Study Design	Campaign Focus	Specific Disease	Campaign Type	Communication Methods	Outcome Measures	Effective
Flemish National Campaign	Bruyndonckx et al. 2020	Belgium	2000-2, 2004-7, 2008-13, 2014-19	Longitudinal	Public	RTI	National	Mass media	Change in public awareness Outpatient antibiotic use Antibiotic cost AMR in specific organisms	Yes
e-Bug	Adriaenssens et al. 2011	Belgium	2006, ongoing	Protocol	Public (children)	NA	International	School curriculum	NA	NA
e-Bug	Herotova et al., 2011	Czech Republic	2006, ongoing	Protocol	Public (children)	NA	International	School curriculum	NA	NA
e-Bug	Hayes et al. 2020	International	2006, ongoing	Protocol	Public (children)	NA	International	School curriculum	How e-bug is promoted, barriers faced by partners	NA
Do Bugs Need Drugs community education programme.	Fuertes et al. 2010	Canada	2005, autumn	Longitudinal	Public	RTI	National	Mass media	Antibiotic consumption rate (DID)	Yes
Do Bugs Need Drugs community education programme.	McKay et al. 2011	Canada	2005, autumn	Longitudinal	Public	RTI	National	Mass media	Change in knowledge and attitudes. HCP knowledge of resistance trends Antibiotic consumption (DDD)	No
Pilot campaign	Kandeel et al. 2019	Egypt	2011, five months	Pilot	Public and HCP	RTI	Community	Social media Site-based resources	Change in knowledge and attitudes Antibiotic prescribing practices	Yes
Public AB campaign	McNulty et al. 2010	England	2008, one month	Longitudinal	Public	RTI	National	Mass media	Change in knowledge, attitudes and behaviour. Effect of delayed antibiotic prescribing	No
Keep Antibiotics Working	UKHSA, 2021	England	2017, three years	Longitudinal	Public	RTI	National	Mass media	Campaign recognition Change in knowledge, attitude and reported behaviour Impact on GP’s	Partially
	Borgonha 2019	England	2017, three years	Mixed-methods					Audience perception of messaging	No
European campaigns and policy change	Filippini et al. 2012	Europe	NA	Pooled analysis	Public and HCP	NA	National	NA	Antibiotic consumption rate (DID)	Yes
French national mass media campaign with local component	Dunais et al. 2011	France	2001-5, 2007-10, 2011-16	Cross-sectional	Public and HCP	RTI	National with local component	HCP education Mass media	Antibiotic prescription data obtained from child health book	Yes
French mass media campaign	Dommergues & Hentgen, 2011	France	2001-5, 2007-10, 2011-16	Cross-sectional	Public	RTI	National	Mass media	Annual antibiotic prescriptions Annual number of consultations	Yes
French mass media campaign	Chahwakilian et al. 2011	France	2001-5, 2007-10, 2011-16	Cross-sectional	Public	RTI	National	Mass media	Antibiotic prescription rates per 1000 inhabitants per year	Yes
French mass media campaign	Carlet et al. 2020	France	2001-5, 2007-10, 2011-16	Cross-sectional	Public	RTI	National	Mass media	Antibiotic consumption in the community Prevalence of resistant infections	Yes
French mass media campaign	Bernier et al., 2014	France	2001-5, 2007-10, 2011-16	Longitudinal	Public	RTI	National	Mass media	Weekly rates of antibiotic prescriptions per 1,000 inhabitants.	Yes
Public education campaign and HCP education	Plachouras et al. 2014	Greece	2009, two months	Quasi-experimental	Public and HCP	Common community infections	Community	Site-based resources HCP interaction	Antibiotic consumption rate (DID)	No
Publicity campaign	Mei Lin Ho et al. 2014	Hong Kong	2011, two months	Longitudinal	Mainly public, some HCP education	RTI	National	Mass media	Change in knowledge and attitudes	Yes
Educational campaign	Khoshgoftar et al. 2021	Iran	2019, one week	Quasi-experimental	Public	RTI	Community	Site-based resources	Change in knowledge, attitudes and self-reported antibiotic consumption	No
Intervention in clinics in Israel	Maor et al. 2011	Israel	2002, two months	Longitudinal	Public (parents)	None	Community	Site-based resources HCP interaction	Change in knowledge and attitude	Yes
Regional campaign	Formoso et al. 2013	Italy	2011, four months	Experimental	Public	RTI	Community	Mass media	Average prescribing rate (DID) Expenditure on outpatient Abs Change in knowledge, attitude and reported behaviour	Yes
Local education programme	Shehadeh et al. 2016	Jordan	2012, four months	Longitudinal	Public	None specified	Local	HCP interaction	Change in knowledge	Yes
National Campaing - “Wise use or lose”	Al-Mousa & Aly, 2011	Kuwait	2009, one week	Protocol	Public and HCP	RTI	National	Site-based resources HCP interaction	NA	NA
School-based education programme	Ngadimon et al., 2016	Malaysia	Not specified	Cross-sectional	Public (children)	None	National	School curriculum	Change in knowledge and attitudes	Yes
Intervention Tool	Thong et al. 2021	Malaysia	Not specified	Longitudinal	Public (parents)	None	Community	HCP interaction	Change in knowledge	Yes
Educational video	van Rijn et al. 2019	Holland	Not specified	Quasi-experimental	Public	None	Community	Educational video	Change in knowledge	No
EAAD campaigns	Mazinska et al. 2017	Poland	2009, run annually	Cross-sectional	Public and HCP	RTI	International	Mass media	Knowledge and attitudes	Yes
Social Media campaign	Zowawi et al., 2015	Saudi Arabia	2013, five months	Pilot	Public and HCP	No	National	Social media	Campaign engagement	NA
Educational campaign “Obiettivo Antibiotico”	Barchitta et al., 2020	Sicily	2018, ongoing	Cross-sectional	Public and HCP	None specified	National	Website-based	Campaign engagement	NA
Education flyers	Furst et al. 2015	Slovenia	1995, ongoing	Quasi-experimental	Mainly HCP with some public focus	RTI	National	HCP education Educational materials	Antibiotic utilisation (DID) Antibiotic resistance	Yes
Community-pharmacy intervention	Munoz et al. 2014	Spain	Not specified	Experimental	Public (patient)	None	Local	HCP interaction	Adherence to treatment	Partially
AB Smart Use Programme	Phueanpinit et al. 2022	Thailand	2007, ongoing	Cross-sectional	Public and HCP	RTI	National	Mass media	Campaign recognition Perceived benefit	NA
Antibiotic Guardian	Kesten et al. 2017	UK	2014, ongoing	Qualitative	Public and HCP	None	International	Website-based	Campaign engagement Behaviour change	No
Antibiotic Guardian	Chaintarli et al. 2016	UK	2014, ongoing	Cross-sectional	Public and HCP	None	International	Website-based	Behaviour change	No
Antibiotic Guardian	Bhattacharya et al. 2017	UK	2014, ongoing	Process evaluation	Public and HCP	None	International	Website-based	Campaign engagement	NA
Antibiotic Guardian	Newitt et al. 2018	UK	2014, ongoing	Cross-sectional	Public and HCP	None	International	Website-based	Campaign engagement AMR knowledge	Yes
Antibiotic Guardian	Newitt et al. 2019	UK	2014, ongoing	Cross-sectional	Public and HCP	None	International	Website-based	Campaign engagement AMR knowledge and behaviour change	Yes
RTI symptom severity intervention	Lawrence and Ferguson 2019	UK	Data collection October 2015 to March 2016	Cross-sectional	Public	RTI	Local	Site-based resources	Severity of RTI symptoms	No
University workshops	Allison et al., 2016	UK	Not specified	Cross-sectional	Public (students)	None	Local	Site-based resources	Perception about AMR percentage of attendees who became Antibiotic Guardians	No
Intervention in Pet owners	Redding and Cole, 2019	USA	Not specified	Cross-sectional	Public	None	Local	Site-based resources	Change in knowledge	No
Educational sessions	Morgan et al., 2021	USA	2018, one year	Quasi-experimental	Mainly HCP, some public (patient) education	None specified	Community	HCP education Site-based resources	Number of antibiotic prescriptions written for antibiotic nonresponsive RTI	Yes

Abbreviations: AMR, antimicrobial resistance; HCP, healthcare professional; RTI, respiratory tract infection

**Table 3:** showing a summary of the key focus and aims of included campaigns and interventions

Authors	Country	Key aims
Bruyndonckx et al. 2020	Belgium	Utilised mass media to disseminate information regarding the natural course of self-limiting infections, appropriate antibiotic usage and the consequence of AMR.
Fuertes et al. 2010	Canada	Annual media campaigns aimed at reducing the number of unnecessary antibiotic prescriptions by educating the public and health care professionals on the appropriate use of antibiotics, primarily focusing on acute upper respiratory tract infections. Key messages included “Wash your hands”, “Antibiotics work against bacteria, not viruses” and “Use antibiotics wisely because bacteria can become resistant to them”.
McKay et al. 2011	Canada	
Kandeel et al. 2019	Egypt	Educational campaign to raise the awareness of physicians, pharmacists, and the general public in the district regarding the importance of rational antibiotic prescribing for acute respiratory infections.
McNulty et al. 2010	England	Use of promotional materials in general practice surgeries and pharmacies to disseminate a key message on self-limiting infections. “The best way to treat most colds, coughs or sore throats is plenty of fluids and rest. For advice talk to your pharmacist or doctor”.
ESPAUR	England	Mass media campaign aimed at improving public awareness, understanding and knowledge of AMR. Also looked to reduced patient demand for antibiotics and improve GP confidence in their prescribing decisions.
Borgonha. 2019	England	
Filippini et al. 2012	Europe	NA
Dunais et al. 2011	France	Mass media campaign was designed to educate caregivers and the public that antibiotics are not always necessary and to describe the appropriate use of antibiotics, with a special focus on respiratory tract infections. The campaign also sought to indirectly decrease antibiotic use by improving vaccine coverage against bacterial diseases.
Dommergues & Hentgen, 2011	France	
Chahwakilian et al. 2011	France	
Carlet et al. 2020	France	
Bernier et al., 2014	France	
Plachouras et al. 2014	Greece	Focus on improving understanding of infectious disease and appropriate antibiotic usage in parents of nursey and primary school aged children.
Mei Lin Ho et al. 2014	Hong Kong	Community-wide publicity campaign to promote safe use of antibiotics focused on the key message that “Antibiotics do not help in cold and flu”.
Adriaenssens et al. 2011	Belgium	Educational resources which aims to reduce the spread of infection and use of antimicrobials in young people and the community, so helping to control AMR.
Herotova et al., 2011	Czech Republic	
Hayes et al. 2020	International	
Khoshgoftar et al. 2021	Iran	Public and HCP campaign to describe antibiotic prescribing practices for respiratory tract infections.
Maor et al. 2011	Israel	Multi-faceted intervention aimed at promoting appropriate antibiotic use. Use of education intervention to promote the notion of parents as partners in the fight against antibiotic resistant bacteria and reduce parental expectation for an antibiotic prescription for their child.
Formoso et al. 2013	Italy	Mass media campaign focused on the use of antibiotics in upper respiratory tract infections and highlighted that antibiotics are necessary in specific circumstances, do not work in case of influenza or colds, and should be used when doctors prescribe.
Shehadeh et al. 2016	Jordan	Pharmacist-initiated educational intervention on participants’ knowledge regarding appropriate and safe antibiotic use, resistance among adults in Jordan, the enhancement of safer antibiotic use and reduction of self-medication.
Al-Mousa & Aly, 2011	Kuwait	Campaign mission was to promote appropriate antibiotic use in healthcare settings through awareness, education, and adoption of best practices. Utilised promotional materials such as leaflets to disseminate information on proper methods for use and the self-limiting nature of colds and flu.
Ngadimon et al., 2016	Malaysia	Educational programme aimed at improving knowledge regarding what antibiotics are, why antibiotics are needed, how to use antibiotics correctly, and how resistance to antibiotics occurs in secondary school students.
Thong et al. 2021	Malaysia	Use of a targeted educational intervention to promote messages on proper antibiotic use, causes of antibiotic resistance, and the responsibilities of the public to stem resistance.
van Rijn et al. 2019	Netherlands	Use of an educational video on the threat of antimicrobial resistance to increase general awareness and determine if uptake of information differs between public campaign target audiences.
Mazinska et al. 2017	Poland	Educational action aimed at the general public.
Zowawi et al., 2015	Saudi Arabia	Use of superbug hashtag on social media to deliver short messages and link to articles and educational videos related to antimicrobial resistance and the importance of antibiotics.
Barchitta et al., 2020	Sicily	Website-based campaign aimed to increase awareness and promote engagement in order and to change behaviour towards the rising threat of AMR in four main categories of target audience: (i) the general public (e.g., adults, parents, and students); (ii) primary care prescribers (i.e., general practitioners and paediatricians); (iii) pharmacists; and (iv) professionals in hospitals and other healthcare settings.
Furst et al. 2015	Slovenia	Multi-faceted campaign, public element provided promotional materials to provide information on responsible use of antibiotics and the management of self-limiting infections, especially in children.
Munoz et al. 2014	Spain	Community pharmacy-based intervention aiming to improve antibiotic adherence and patient-reported resolution of symptoms.
Phueanpinit et al. 2022	Thailand	Aimed to educate the public in appropriate antibiotic use for upper respiratory tract infections.
Kesten et al. 2017	UK	Website-based campaign aimed to increase commitment to reducing antimicrobial resistance, change behaviour and increase knowledge through an online pledge system for healthcare professionals and members of the public to become Antibiotic Guardians. Originally launched in the UK it has become a global cmapaign.
Chaintarli et al. 2016	UK	
Bhattacharya et al. 2017	UK	
Newitt et al. 2018	UK	
Newitt et al. 2019	UK	
Lawrence and Ferguson 2019	UK	Assessing impact of messaging on non-antimicrobial means to treat RTIs (e.g. paracetamol, fluids, rest), the ineffectiveness of antibiotics in the treatment of RTIs (colds and flu), antibiotic side effects (including diarrhoea, thrush), and the link between the over-use of antibiotics and AMR.
Allison et al., 2016	UK	Promote knowledge about antibiotic resistance development and good stewardship principles amongst the general population through pharmacy student-led public engagement workshops in high schools.
Redding and Cole, 2019	USA	Use of posters to promote antimicrobial stewardship messages related to upper respiratory tract infections in cats and dogs.
Morgan et al., 2021	USA	Use of a behaviourally enhanced quality improvement intervention to reduce the number of antibiotic prescriptions written for antibiotic nonresponsive acute respiratory infections.

Abbreviations: AMR, antimicrobial resistance; GP, general practitioner; HCP, healthcare professional; RTI, respiratory tract infection

### Study design

The majority of studies used cross-sectional [18, 21, 27, 29, 30, 34, 35, 37, 40, 42, 45, 47, 50–53] or longitudinal study designs [19, 20, 22, 24, 38, 39, 44, 46, 53, 54], (*n* = 14 and 10 respectively). five used a quasi-experimental [25, 32, 33, 36, 49] and two used an experimental design [23, 56]. 

### Target population

The primary focus of 17 of the campaigns was the public [19, 21, 23, 24, 30, 33, 35, 38, 46, 49, 55], with two school curriculum-based campaigns focused on children [26, 27, 42, 47], two further campaigns focused on parental education [20, 44], and one campaign focused on patients [56]. The remaining 12 campaigns targeted both HCP and the public [18, 22, 25, 28, 29, 31, 32, 36, 45, 48, 57, 58]. 

### Campaign type and communication methods predominantly used

Most were national campaigns (*n* = 13) which used mass media to disseminate information (*n* = 7) [19, 21, 22, 24, 38, 39, 45]. Other national campaigns used the school curriculum [47], social media [31], websites [29, 37], and interactions with HCP combined with education resources [28, 36] to communicate with their target audiences. Eight studies evaluated community or regional-level campaigns, with site-based resources, such as poster displays and leaflets, the most common communication method (*n* = 5) [20, 25, 32, 48, 49]. Local campaigns used HCP interaction (*n* = 2) [46, 56] and site-based resources, such as posters (*n* = 3) [30, 35, 40]. Finally, three international campaigns were identified which used mass media [58], school curriculums [26, 27, 42], and website content [37] to disseminate information.

### Outcome measures

Change in participants knowledge of and/or attitudes towards AMR was predominantly used as an outcome measure (*n* = 16) [22, 23, 30, 33, 34, 38, 39, 43, 46–51, 55, 59]. Trends or changes in antibiotic prescribing or usage was also commonly used (*n* = 14) [18, 19, 21, 23–24, 32, 36, 48, 52–55, 60]. Other outcome measures used included reported behaviour change (*n* = 8) [23, 37–39, 49, 51, 56, 57], campaign engagement (*n* = 6) [29, 31, 50, 51, 57, 61], expenditure on antibiotics (*n* = 2) [19, 21], annual number of GP consultations (*n* = 1) [21], severity of respiratory tract infection symptoms (*n* = 1) [35], and perception of campaign messaging (*n* = 1) [62]. 

### Changes in knowledge, attitudes, and behaviour

Table 4. summarises studies which assessed changes in knowledge, attitudes and behaviour following the campaign. Studies reviewing the pledge-based Antibiotic Guardian campaign assessed self-reported change in knowledge which shows 44·5% of Antibiotic Guardians reported an increase in knowledge post-campaign. This was more likely in individuals with limited pre-campaign knowledge of AMR (OR: 4·21, CI: 2·04–8·67) [37]. Furthermore, when making a pledge, Antibiotic Guardians were asked five knowledge-based questions. Comparison of these to Eurobarometer questionnaire responses by the UK or EU public showed Antibiotic Guardians answered more questions correctly (OR: 8·5 and 13·9 respectively) [51, 63]. However, not all studies analysing the Antibiotic Guardian campaign reported positive findings only. A qualitative analysis by Kesten et al. showed that a limited number of Antibiotic Guardians could fully recall their pledge and reports regarding improved knowledge following participation in the campaign were mixed [57]. 

**Table 4:** Summary of key findings from studies assessing changes in knowledge, attitudes and or reported behaviour as key outcome measure

Author	Country	Study Design	Intervention	Sample	Year of intervention	Campaign focus	Campaign Type	Comparator	Outcome Measure	Findings
Mei Lin Ho et al. 2014	Hong Kong	Longitudinal	Six months of mass media messaging with key message ‘Abs do not help in cold and flu’. Information resources also available in hospitals and clinics. Promotional materials also provided to HCPs	*n* = 1569 (response rate 69.3%) in Nov 2010 *n* = 1527 (response rate 66.6%) in June 2011. Random sample of members of the public aged > 18yrs	2011	Mainly public, some HCP education	National	Pre and post campaign	Knowledge and attitudes related to AMR and antibiotic use	27.7% of respondents could recall the campaign, 73.9% through TV advertising. Increase in number of individuals correctly identifying that antibiotics do not cure flu or viral infections (7, *p* < 0.001, and 4.1%, *p* = 0.002, increases respectively)
Formoso et al. 2013	Italy	Experimental	Social marketing information campaign between Nov 2011 and Feb 2012 in Moderna and Parma. Focused on the use of Abs in URTI.	600 citizens in the intervention and control areas were randomly selected. 500 were interviewed by phone, 100 by internet	2011 and 2012	Public	Community	Pre and post campaign survey	Knowledge, attitudes and reported behaviour about the campaign messages	Control and intervention groups had similar baseline scores. Following the campaign, only knowledge on the effectiveness of antibiotics against viruses changed significantly, worsening more in the intervention compared to the control area (‘Antibiotics are effective against viruses’ pre: intervention = 47%, control = 59%; post: intervention = 62%, control = 67%).
Redding and Cole, 2019	USA	Cross-sectional	Posters showing antimicrobial stewardship messages related to RTI in dogs and cats were placed in clinic exam rooms for a 6-month period	Five privately owned veterinary clinics. 111 participants, 91 completed a follow up survey with 83 providing a definition of antibiotic resistance	Unclear, paper published in 2019	Public	Local	NA	Survey assessed message recall and knowledge related to AMR.	51 (46.4%) of participants noticed the posters within the clinic. 15.7% (8/51) participants paraphrased the message and 5.9% (3/51) reproduced the message. No significant difference in knowledge score between those who had and had not noticed the posters (*p* = 0.693)
van Rijn et al., 2019	Netherlands	Quasi-experimental	Educational video explaining what antibiotics are, how bacteria become resistant to antibiotics and the importance of prudent use of antibiotics. Study aimed to determine the effect of this information on the general awareness of antibiotic resistance.	Control group: *n* = 560 Intervention group: *n* = 1220	Unknown	Public	Community	Control group who was not shown the educational video	Level of knowledge. The number of correct knowledge-based questions for each individual was summed and categorised into three groups - Group 1: low knowledge < 4 correct answers (*N* = 332, 18.7%); group 2: moderate knowledge 4 to 7 correct answers (*N* = 1024, 57.5%); Group3: highest knowledge > 7 correct answers (*N* = 424, 23.8%).	Educational video raised general awareness of AMR in the intervention group (*p* = 0.048) with 67% of the intervention group had a better general awareness of antibiotic resistance. Among the lowest knowledge group, those in the intervention group had a significantly higher mean score on the general awareness of the threat of antibiotic resistance than the control group (*p* < 0.001). 67% of the intervention group had a better general awareness of antibiotic resistance.
Mazinska et al., 2017	Poland	Longitudinal	EAAD campaign with intensive educational action aimed at the public. Activities included press conferences, posters, leaflets for patients, leaflets for children and parents, exhibition displayed nationwide (16 posters), 10 s. spot broadcast in public TV, 10 s, 30 s. spots in public TV, Facebook since 2011.	5004 respondents were recruited: 1000 in wave 1 (October 2009), 1002 in wave 2 (December 2009), 1001 in wave 3 (October 2010), 1000 in wave 4 (December 2010) and 1001 in wave 5 (December 2011). The response rates were 5.1 -11.3% (2009–2011).	2009 to 2011	Public and HCP	International	Pre and post data collected before and after the EAAD campaigns run in 2019 and 2010 and following the campaign in 2011.	Sources of knowledge Knowledge and impact of the campaign on attitudes towards the use of antibiotics	One third of the respondents (29%) declared that they received information about antibiotics. Fewer of the respondents had used antibiotics to treat colds (2009: 30%, 2011: 21%, *p* = 0.008), sore throats (2009: 24%; 2011: 12%, *p* < 0.001) and flu (2009: 16%; 2011: 13%,*p* = 0.124). More respondents declared they had taken the full dose of antibiotic prescribed (2009: 75%, 2011: 81%, *p* = 0.063) Fewer respondents expected a prescription for antibiotics because of colds (2009: 19%; 2011: 15%, *p* = 0.019) and flu (2009: 43%; 2011: 32%, *p* < 0.001). More respondents declared a change in behaviour after receiving EAAD campaign information (2009: 38%; 2011: 48%, *p* = 0.012). More respondents who came across the campaign indicated they have limited the use of antibiotics (2009: 27%; 2011: 43%, *p* = 0.009) and they have become more cautious regarding the use of antibiotics (2009: 4%: 2011: 16%, *p* = 0.001)
UKHSA., 2021	England	Longitudinal	National, mass media, social marketing campaign. The direct-to-public communications were supplemented with resources that prescribers could download and customise for their surgeries.	Random sample of the public. December 2017: *n* = 1201; December 2018: *n* = 1352; December 2019: *n* = 1350. Purposive sampling in target groups (mothers of children aged 0–16 and adults over 50). GPs: 2017, *n* = 189; 2018, *n* = 172; 2019, *n* = 207	2017 to 2019	Public	National	Pre and post campaign comparison for each year the campaign was run.	Campaign recognition Knowledge, attitudes and reported behaviour	Campaign recognition increased each year (56%, 65% and 71%). Knowledge increased for AMR specific questions including ‘Antibiotics will stop working if taken for the wrong things’: 2017, 29%; 2018, 33% and 2019, 35% answered definitely true; ‘Taking antibiotics when you don’t need them puts you and your family at risk of antibiotic resistant infections’: 2017, 36%; 2018, 45 and 2019, 48% answered definitely true). Concern increased for self and for child with percentage very unlikely to ask for AB for self (2017, 53%; 2018, 53%; 2019, 48%) or for child (2017, 38%; 2018, 34%; 2019, 33%) decreasing each year.
McNulty et al., 2010	England	Longitudinal	Public campaign using posters and patient advice leaflets to disseminate information	1706 English and 182 Scottish adults in January 2008; 1707 English and 123 Scottish adults in January 2009. Random location sampling and non-random quota sampling used.	2008	Public	National	Pre and post comparison	Publics knowledge, attitudes and behaviour (reported AB use) with respect to AB use.	Increase in percentage of individuals that recalled any of the three campaign posters (19.2 to 23.7%). Absolute change from baseline (including both England and Scotland response) was 2.3%. Respondents were more likely to agree with the statement ‘Resistance to ABs is a problem in British hospitals’ (37% vs. 30%, *p* = 0.03). Reported antibiotic use did not improve. A similar percentage of English respondents reported being prescribed an antibiotic between 2008 and 2009 (34 vs. 35%) or reported asking their GP for an antibiotic between 2008 and 2009 (28 vs. 29%). The percentage of English respondents retaining leftover ABs from their last course increased following the campaign (2.2 vs. 7.0%, *p* < 0.001).
Thong et al., 2021	Malaysia	Longitudinal	Educational intervention tool developed after reviewing knowledge gaps. Each educational session was conducted by trained pharmacists and lasted approximately 15 min for each participant.	300 recruited and 234 respondents who completed the study were included in the final analysis.	Unknown	Public (patients)	Community	Pre and two weeks post intervention comparison	Knowledge on antibiotic resistance and use. Perception of antibiotic use.	Knowledge of antibiotic resistance: number of respondents answering the statement “Taking leftover antibiotics from previous infection may cause antibiotic resistance” increased by 26.1% after the intervention (64.1 to 90.2%, *p* < 0.001). Correct responses to “Using antibiotics without consulting doctor or purchasing directly from the pharmacy without a prescription may cause antibiotic resistance” increased by 19.2% (69.7 to 88.9% *p* < 0.001). Knowledge on antibiotic use: increase in number of respondents that understood antibiotics cannot treat viral infections (25.6 vs. 80.3%, *p* = 0.004). Improvement in the number of respondents who could correctly identify conditions treatable with ABs (9.8% vs. 43.2%, *p* = 0.022). Perception of antibiotic use: Improvement in respondents’ belief they had a role to play in stopping AMR (26.5 to 69.2%, *p* < 0.001). Increase in concern about the impact of AMR on their health and their family (66.2 vs. 86.8%, *p* < 0.001). Improvement in all correct responses to questions relating to respondents’ perception on antibiotic use was seen following the intervention (*p* < 0.001).
Shehadeh et al., 2016	Jordan	Longitudinal	Participant was verbally educated on one-to-one basis using educational card contained information based on the published educational materials by the Centre for Disease Control and Prevention. Additionally, the educational card included further information outlined in the previously published recommendations	271 adults completed both questionnaires (purposive sampling)	April to July 2012	Public	Local	Pre and post questionnaire	Knowledge about appropriate antibiotics use and resistance.	Knowledge scores improved following the intervention (pre: 59.4; post: 65.9% *p* < 0.001). Knowledge of appropriate use (i.e. not effective against viral infections) improved (Pre: 45.8%; post: 75.9%, *p* = 0.035). Awareness regarding antibiotic use by pregnant, lactating mothers and young children increased (pre: 17.6, post: 81.3%; pre: 31.1, post: 77.9%; pre: 52.7, post: 63.7%). The knowledge score (SD) for safe use of antibiotics increased (pre: 23.8% (9.7), post: 76% (26.7), *p* = 0.017).
Ngadimon et al., 2016	Malaysia	Longitudinal	20-30 min antibiotic education session delivered verbally to students based on education content from the National Pharmaceutical Control Bureau, Ministry of Health, Malaysia.	510 secondary school students (13 to 17 years). Schools were randomly selected.	Unknown	Public (students)	National	Pre and post comparison	Antibiotics knowledge and attitude toward antibiotics	Mean knowledge (Pre: 3.2 ± 1.8 vs. post: 6.8 ± 2.1, *p* < 0.001) and attitude scores (Pre: 3.3 ± 1.7 vs. post: 5.4 ± 1.9, *p* = 0.003) increased significantly following the intervention. Positive correlation between attitude and knowledge scores (0.4, *p* < 0.001).
Kandeel et al., 2019	Egypt	Pilot	Campaign aimed to raise awareness of HCP and the public of the importance of rational antibiotic prescribing for RTIs. Social media campaign targeted youths. Training course provided to HCP and fact sheets on RTI management guidelines. Posters displayed in GP waiting areas, pharmacies, community areas, schools and universities.	Randomly selected patients from acute care hospitals and a sample of primary healthcare centres in Minya. Pre- intervention: *n* = 113; post-intervention: *n* = 277	2011	Both	Community	Pre and post comparison	Clinician and public attitudes and knowledge Self-reported antibiotic prescribing practices	Overall knowledge and attitude scores increased among caregivers and adult patients after the intervention (from 2.3 to 2.5 and from 2.4 to 2.6, respectively). Self-reported prescribing practices: the percentage of physicians who reported prescribing antibiotics most of the time for colds, bronchitis, sinusitis decreased following the intervention (9.3 to 2.1%, 65.8 to 28.4% and 43.5 to 17.0% respectively). Percentage of pharmacists prescribing antibiotics or recommending antibiotics for RTI (83.6 to 57.7%, 57.8 to 24.8%) and in the percentage of pharmacists prescribing antibiotics for colds declined (67.5 to 28.0%).
Khoshgoftar et al., 2021	Iran	Quasi-experimental	Educational campaign using informative and persuasive style messaging developed to disseminate info on resting during colds, ineffectiveness of Abs at treating viruses, the effects of arbitrary antibiotic use and the need to prescribe medication.	Random sampling in ten urban areas (*n* = 708)	Unknown	Public	Community	Pre and post comparison	Knowledge, attitude and self-reported antibiotic consumption (behaviour change).	Mean knowledge (23.27 vs. 38.96) and attitude (15.00 vs. 25.61) increased after the intervention (*p* < 0.001).
Newitt et al., 2018	International	Cross-sectional	Behaviour change and engagement campaign - Antibiotic guardian. Online pledge-based system designed to improve knowledge and behaviours regarding AB prescribing and use among HCP and the public. 2-min video aimed at the public, promotion of key messages via social media, resource development and toolkits for HCP, development of an interactive quiz.	Antibiotic guardian website visitors. Members of the public: *n* = 885	Ongoing	Both	International	Pre- post translation comparison.	Antimicrobial resistance knowledge	94.2% of questions were answered correctly (16,918/17,965). Russian pages had the lowest proportion of correct responses (78.5%, *n* = 208) compared to Dutch (93.9%, *n* = 1179) and English pages (94.4%, *n* = 15,531). A higher proportion of Antibiotic Guardians answered all four Eurobarometer survey questions correctly compared to the published results of the EU group (80.9% vs. 24%, *P* < 0.001). AG’s who were members of the public were also more likely to answer all four questions correctly compared to the published results of the EU group (70.2% vs. 24%, *P* < 0.001).
Newitt et al., 2019	International	Cross-sectional	Behaviour change and engagement campaign - Antibiotic guardian. Online pledge-based system designed to improve knowledge and behaviours regarding AB prescribing and use among HCP and the public. 2-min video aimed at the public, promotion of key messages via social media, resource development and toolkits for HCP, development of an interactive quiz.	3289 Antibiotic Guardian pledgers answered questions on AMR knowledge and 1940 AG’s completed online survey on self-reported behaviours	Ongoing	Both	International	Euro-barometer survey data on AMR knowledge	Self-reported knowledge and behaviour change	80% of Antibiotic Guardians answered all 5 questions correctly. For individual questions, the percentage that were correctly answered ranged from 91.1–98%. HCP answered more questions correctly than the public (96.5 vs. 91.2%, *p* < 0.001). Antibiotic Guardians answered more questions correctly when compared to UK (OR 8.5) and EU (OR 13.9) public. Antibiotic Guardians answered an average of 3.8 questions correctly compared to 2.8 and 2.5 for UK and EU public respectively.
McKay et al., 2011	Canada	Longitudinal	Do Bugs Need Drugs (DBND) programme had two main arms. Public component which included annual media campaigns, print material distribution, and audience-specific education curricula targeting children. The HCP education offered accredited courses to physicians and pharmacists, with a focus on antibiotic use, resistance, and strategies to prescribe appropriately	63 physicians who completed the accredited continuing education course for physicians in 2008	2005	Both	National	Pre and post analysis	Public knowledge and attitudes assessed via telephone survey before and after the initial launch of the programme. Physician and pharmacist knowledge of resistance trends, aetiology of respiratory infections and treatment options assessed via pre- and post-course learner assessments.	Assessment of public knowledge not evaluated due to low response rates (pre: 14.9% and post: 18.3) GP knowledge - knowledge scores for bronchitis (70.35 vs. 81.43%), otitis media (66.84 vs. 85.16%), sinusitis (67.46 vs. 70.85%) and pharyngitis (73.33 vs. 90.16%) increased. An assessment of general knowledge about antibiotics and resistance showed an improvement in correct responses after the course (mean increase 11.2%; *P* = 0.013)
Maor et al., 2011	Israel	Longitudinal	Educational intervention aimed at parents accompanying children to participating clinics. Aimed to enhance knowledge regarding antibiotic resistance and it major determinant, non-judicious antibiotic use and decrease parents expectations to receive an antibiotic prescription.	1556 parents, 868 pre-campaign (control group) and 688 post campaign.	2002	Public (parents)	Community	Pre and post comparison	Knowledge of appropriate antibiotic use	Individuals who noticed the campaign has higher odds for low expectation for antibiotics (OR 1.51, *p* < 0.001). Intervention group had good knowledge (classed as scoring > 50% on test) about AB treatment compared to parents in the control group (45.1 vs. 36.8%, *p* = 0.005). Intervention group had a stronger tendency to avoid unnecessary AB treatment compared to the control group, (53.2% vs.59.9%). Similarly, higher proportion of intervention group did not expect antibiotic treatment on the survey day (48.6% vs. 56.7%, *p* = 0.001).

Abbreviations: n = sample size; AMR, antimicrobial resistance; GP, general practitioner; HCP, healthcare professional; RTI, respiratory tract infection

Multiple campaigns used a pre- and post-campaign comparison of a knowledge-based questionnaire to determine the campaign’s effectiveness. Ho et al. showed a significant increase in knowledge regarding the ineffectiveness of antibiotics at treating viral infections following a mass media campaign promoting the key message “Antibiotics do not help in cold and flu” [22]. Thong et al. and Shehadeh et al. assessed the effect of one-to-one educational sessions conducted by HCPs; both showed significant improvements in knowledge of antibiotic use and resistance [44, 46]. Another intervention using a taught component delivered education sessions to school children as part of the school curriculum in Malaysia [47]. Assessment of knowledge showed a significant improvement in both mean knowledge and attitude scores as well as a correlation between the two scores. Finally, using independent samples, Mazinska et al. evaluated the impact of European Antibiotic Awareness Day (EAAD) in Poland [34]. Questionnaire data was collected from a random sample of adults aged over 18-years pre and post EAAD in 2009, 2010 and 2011 and showed those who were aware of the EAAD campaign (29·0% of respondents) had significantly better knowledge of appropriate antibiotic usage than those who were not aware of the campaign, especially regarding the use of antibiotics to treat RTIs [34].

A British public antibiotic campaign launched in 2008 was also evaluated using pre- and post-campaign questionnaire data [39]. While recognition of campaign materials, such as posters, increased over the campaign period, there was no significant change in attitudes, including attitudes towards the use of antibiotics to treat colds and flu despite this being the campaign’s key message. Furthermore, no significant change in self-reported antibiotic use occurred following the campaign. However, the latter Keep Antibiotics Working campaign showed significant improvements in some aspects of public knowledge [43]. Campaign recognition was higher than for the 2008 campaign (23·7 vs. 71·0%) and knowledge increased significantly for AMR specific questions such as “antibiotics will stop working if taken for the wrong things”. Levels of concern about AMR within the public also increased significantly post-campaign.

### Changes in antibiotic prescribing

Table 5. summarises findings from studies which used antibiotic prescribing rates as their primary outcome measure. Studies that determined the effectiveness of the 2002 French mass media campaign used antibiotic prescribing data, obtained from several sources including prescribing panels, sales data from IMS Health France, government organisations, and National Insurance reimbursement data, as a primary outcome measure [21, 52–54]. These studies indicated a significant reduction in antibiotic prescribing with reductions of up to 33·0% during the final campaign period from 2009 to 2010. This effect however was not consistent across age groups with greater reductions seen in children compared to older adults, which is thought to be due to the introduction of the pneumococcal conjugate (7-valent) vaccine for children aged less than 5-years old [54]. Furthermore, Dunais et al. also analysed a local intervention which commenced in 2001 and ran alongside the national campaign until 2003 [18]. This local intervention was focused on GPs and paediatricians and aimed to improve the management of RTIs in children under the age of 6-years. Analysis of antibiotic prescribing data obtained from children’s health books showed a 50·0% reduction in the number of children receiving antibiotic prescriptions between 1999 and 2008. This reduction occurred despite no significant change in the average weekly number of RTI and bronchiolitis cases reported by GPs. Dommergues & Hentgen also found a 50·4% reduction in national annual antibiotic prescriptions within children aged under 18-years from 2001 to 2010, with the greatest reduction seen in children aged 0 to 24 months (57·2%) [21]. Although antibiotic prescribing rates have stabilised in recent years, Carlet et al. observed antibiotic prescribing rates remained 12·6% below baseline levels 14-years post-campaign [53]. 

**Table 5:** Summary of key findings from studies assessing changes in antibiotic prescribing and/or usage as key outcome measure

Author	Country	Study Design	Intervention	Comparator	Outcome Measure	Findings
Dunais et al. 2011	France	Cross-sectional	Local intervention aimed at GPs and paediatricians focused on management of uncomplicated RTI’s in children under the age of 6-years. Academic detailing sheets with guidelines were distributed to over 90% of targeted GPs. Run concurrently with National campaign which began 2002.	NA	Antibiotic prescription data. Obtained from child health book or parental questionnaire if health book was not available	Number of children treated with an antibiotic decreased by 50% between 1999 and 2008 with no significant change in the number of average weekly cases of RTI and bronchiolitis reported by GPs between October to April in children aged less than 5 across the three study periods (2.1, 2.4 and 2.2 cases/physician/week respectively).
Dommergues & Hentgen, 2011	France	Cross-sectional	National awareness campaign implemented in November 2002 to reduce inappropriate antibiotic use, especially for RTIs in children aged 0-6yrs. Campaign was repeated every winter from 2002.	NA	Annual estimate of prescriptions Annual number of consultations	Number of GP consultations decreased from 14.1 million in 2001 to 10.4 million in 2010. The proportion of consultations leading to antibiotic prescription decreased from 20.1–26.0% in the 2000–2004 period to 13.6–14.6% in the subsequent years. The overall annual number of antibiotic prescriptions in children decreased by 50.4% with greatest reductions in prescribing seen for upper respiratory tract infections.
Chahwakilian et al. 2011	France	Cross-sectional	Yearly mass media campaign since autumn 2002. National campaign aiming to inform the public about appropriate ambulatory antibiotic use, with a focus on RTIs. The public campaign was complimented by interventions targeting prescribers.	NA	Antibiotic prescription rates per 1000 inhabitants per year	21.9% decline in prescriptions per 1000 inhabitants per year (PIY) between 2001 and 2004 which plateaued following 2004. Attributable to a decreasing in primary-care prescribing for RTIs (-39.9%). Decrease was most significant in children < 15 years. Prescriptions per 1000 children per year decreased by 32.1% from 2001 to 2004 and by 15.8% from 2004 to 2009 compared to a 31.1% decline in 15–64-year-olds and a 10.7% decline in > 65 year-olds between 2001 and 2009. Proportion of consultations for RTIs resulting in antibiotic prescriptions decreased from 58% in 2001 to 44% in 2004. This decline was not maintained after 2004.
Formoso et al. 2013	Italy	Experimental	Social marketing information campaign between Nov 2011 and Feb 2012 in Moderna and Parma. Focused on the use of antibiotics in RTI.	Control group were provinces in Emilia-Romagna where no campaign had been implemented	Average prescribing rate of antibiotics to outpatients during a 5-month period expressed as DIDs. Change after five months in expenditure on outpatient antibiotics per 1000 inhabitants/day.	During follow-up, average prescribing rates decreased by 11.9 and 7.4% in intervention and control regions respectively. A significant difference (4.3%) in reduction of antibiotics prescribed in the intervention areas compared with control areas was seen A 3.2% decrease was seen in the rest of Italy. Expenditure on antibiotics reduced by 25.1 and 21.8% compared with the same period in the previous year in intervention and control groups respectively.
Bruyndonckx et al. 2020	Belgium	Longitudinal	Mass media national awareness campaign aimed at providing the public with a better understanding of the natural course of self-limiting antibiotics, the consequence of AMR, and at facilitating discussion between patients, clinicians, and pharmacists. Key messages changed annually	Pre-campaign data	Outpatient antibiotic use, AMR and cost. Outpatient antibiotic use between 1997 and 2018. Cost of antibiotic use	DID had reduced by 12.8% from 1999–2000 to 2017-18. Cost of antibiotics decreased by 65.3% from 1999–2000 to 2017-18.
Carlet et al. 2020	France	Longitudinal	Yearly mass media campaign since autumn 2002. National campaign aiming to inform the public about appropriate ambulatory AB use, with a focus on RTIs. Interventions also targeted prescribers.	NA	Antibiotic consumption in the community (prescriptions per 1000 pop per year).	Initial notable decrease following the first public health campaign, antibiotic consumption in 2016 was 12.6% lower than in 2000. Most notable change was in children who had a far larger reduction (from 2111 to 1000 prescriptions/1000 pop/year in 2000 and 2014).
Fuertes et al. 2010	Canada	Longitudinal	“Do Bugs Need Drugs” community education programme. Started in January 2006 with initial television campaign. Key messages included wash your hands, antibiotics work against bacteria not viruses, use antibiotics wisely because bacteria can become resistant to them.	Pre (January 1996 to December 2005) and post (January 2006 to December 2008)	Drug utilisation data, antibiotics dispensed through community pharmacy. Antibiotic utilisation rates for adults (defined daily doses per 1000 pop per day) and children less than 15 years of age (prescriptions per 1000 population per day).	During the three years following program implementation, cumulative observed antibiotic use was 5.8% lower than expected. The observed number of cumulative prescriptions dispensed to children was 10.6% lower than expected.
McKay et al. 2011	Canada	Longitudinal	“Do Bugs Need Drugs” community education programme. Started in January 2006 with initial TV campaign. Key messages included wash your hands, antibiotics work against bacteria not viruses, use antibiotics wisely because bacteria can become resistant to them.	Pre and post analysis.	Prescription pads used with patients with RTI symptoms. Monitoring overall antibiotic consumption (1996 to 2008) (Antibiotic consumption rates were expressed in DID).	Significant decrease in use of antibiotics for acute bronchitis (34.6% vs. 21.4%; *P* = 0.023), and for all indications (45.6% vs. 39.2%; *P* = 0.019). Consumption of all antimicrobials reached its lowest level in 2002. The rate of use increased between 2002 and 2005, levelling off since 2006. Prior to the programme, the consumption rate for acute otitis media was declining and continued to decrease following implementation of the program. Between 2002 and 2005, DID rate for acute bronchitis increased from 0.68 to 0.94 (39.1%) DID.
Bernier et al., 2014	France	Longitudinal	Nationwide public health campaign was launched (“Antibiotics Are Not Automatic!” and “Antibiotics, Used Unnecessarily, Lose Their Potency!”). Repeated annually from October to March since 2002, aimed to decrease prescriptions in the community, particularly for children	“campaign” period (October to March), coincides with the targeted public service campaign, compared to the second, the “warm” period, corresponds to April to September	Aggregated 2000–2010 data on all outpatient antibiotics prescribed. Data presented as weekly rates of antibiotics prescriptions per 1,000 inhabitants.	Greatest decrease in prescribing during campaign periods was seen from 2006-7 (30%). After this, reductions were smaller (25 to 27%) but still significant compared to baseline. Similar trend found in children from all age groups (0-5-years: -33%; 6-15years: -24%) and adults (16–60 age group) (-17%), *p* < 0.0001. Trend differed for older adults (> 60-yrs) with fluctuations around baseline values and only two significant decreases, with the largest being observed in 2006 to 2007 (− 9.0% [95% CI: −14.9, − 3.2%]; *P* = 0.004).
Kandeel et al. 2019	Egypt	Pilot	Campaign aimed to raise awareness of the importance appropriate prescribing. Social media campaign and posters displayed in GP waiting areas, pharmacies, community areas, schools and unis. Training and RTI management guidelines for HCPs.	Pre and post	Self-reported antibiotic prescribing practices antibiotic prescribing practices	25% decrease overall in antibiotic prescribing post-intervention for children and a 22% decrease in prescribing for adults which were mainly driven by reductions in prescribing for ear infections and bronchitis. The percentage of physicians who reported commonly prescribing antibiotics for RTIs decreased following the intervention (colds: 9.3 to 2.1%, bronchitis: 65.8 to 28.4% and sinusitis: 43.5 to 17.0%). The percentage of pharmacists prescribing AB (83.6 to 57.7%) or recommending antibiotics (57.8 to 24.8%) for ARI also declined.
Filippini et al. 2012	Europe	Pooled analysis	Countries that adopted some policy measures.	Countries that did not introduce any policy instrument to reduce antibiotic consumption	Consumption rate of antibiotics expressed as DID.	Implementation of a public campaign may reduce antibiotic consumption by 1.3–5.6 DID. This represents an impact of roughly 6.5–28.3% on the mean level of antibiotic use in Europe between 1997 and 2007.
Furst et al. 2015	Slovenia	Quasi- observational	Educational materials provided to the public including flyers on ‘safe use of drugs’, ‘My child has fever’, ‘Get well without Abs’, ‘Interactions of drugs’. Other interventions aimed at HCP include prescribing restrictions workshops, budget targets etc.	Pre and post	AB utilisation. AB resistance	From 1999 to 2012, antibiotic use decreased by 31%, following a 24% increase from, 1995 to 1999. Within the time period 1999–2012, the consumption of antibiotics with prescribing restrictions was reduced on average by 42% and that of non-restricted ones was reduced by 15%. Between 1999 and 2012, the penicillin resistance in invasive S. pneumoniae isolates decreased from 14.5 to 10%. The resistance of S. pneumoniae to macrolides increased from 5.4 to 21%. The resistance of E. coli to fluoroquinolones continuously increased from 10 to 21%.
Plachouras et al. 2014	Greece	Quasi-experimental	Public education campaign and academic detailing of the primary care physicians in the district of Corinth. Seventeen two-hourly educational meetings were organized with parents of children in nursing care and primary school. Following this, parents were given an educational pamphlet on the use of antibiotics for common infections in the community.	The rest of districts in Peloponnese and the national rate served as controls. Data was compared before and after the campaign.	Antibiotic consumption data, antibiotics prescribed for RTIs were included in the study	Antibiotic utilization in the test region (Corinth) was unchanged in January and February 2009 at 26 DID and increased to 32 DID in March 2009, reflecting control region trends.
Morgan et al., 2021	USA	Quasi-experimental	Educational sessions, sharing of data by clinics, and patient and physician educational materials. The intervention period began with resident education and staff meetings.	Pre post comparison using time series analysis.	Number of antibiotic prescriptions written for antibiotic nonresponsive RTI	Average of 166 eligible visits per month. The percentage of visits that resulted in a prescription for an antibiotic unresponsive RTI reduced from 11.5 to 5.8%. Immediate intervention effect indicated a 46% reduction in antibiotic nonresponsive ARI antibiotic prescriptions or 0.54 times (95% CI, 0.42–0.66; *P* = 0.001) as many antibiotic nonresponsive RTI antibiotics prescribed after the intervention. Long term intervention effect showed a statistically significant trend in the number of antibiotic-nonresponsive RTI antibiotics prescribed across all clinics

Abbreviations: AMR, antimicrobial resistance; DDD, defined daily dose; DID, defined daily dose per 1000 inhabitants per day; GP, general practitioner; HCP, healthcare professional; RTI, respiratory tract infection

The Belgium mass media campaign also used antibiotic prescribing data (defined daily doses per 1000 inhabitants per day (DID)) to show a significant impact from the campaign [19]. Following an initial decline of 12·1% from baseline to the end of the second campaign wave in 2007, prescribing rates fluctuated. However, the reduction in prescribing was maintained at 12·6% in 2018.

A pilot campaign in Egypt which aimed to raise awareness of rational antibiotic prescribing for RTIs among physicians, pharmacists, and the public used training courses to educate HCPs and social media to disseminate information within younger populations [48]. A 25·0 and 22·0% decline in antibiotic prescribing was seen post-intervention for children and adults respectively, with changes mainly being driven by reductions in prescribing for ear infections and bronchitis.

Studies evaluating the Canadian “Do Bugs Need Drugs” campaign reported inconsistent effects on antibiotic prescribing. Cumulative observed antibiotic use during the three years following the campaign implementation was 5·8% lower than expected values, with greater effects seen in the number of prescriptions dispensed to children (-10·6%) [23]. Furthermore, the effect on prescribing rates was dependent on the type of antibiotic and the condition for which the antibiotic was being prescribed [55]. 

Studies by Formoso et al. and Plachouras et al. used similar quasi-experimental study designs with campaigns implemented in specific regions with neighbouring provinces and national rates used as controls [23, 32]. Formoso et al. focused on antibiotic use in RTIs and used social marketing strategies to develop campaign messaging. Messaging was then disseminated via local media channels. Analysis of regional outpatient prescribing databases showed a significant 4.3% (95% CI: -7.1 to -1.5%) reduction in prescribing (DID) in intervention compared to control areas. Plachouras et al. relied on two-hourly education sessions for parents conducted by local HCPs and had limited use of the media to promote information. Within the Greek test regions antibiotic consumption was unchanged following the intervention and continued to follow trends seen nationally and within control regions.

A Slovenian campaign predominately aimed at HCP through educational workshops and the implementation of prescribing guidelines and restrictions, but with an element of public education in the form of flyers and posters on topics such as “The safe use of drugs” and “Get well without antibiotics” was implemented in 1995 [36]. Following an initial increase in prescribing rates of 3·97 DID from 1995 to 1999, antibiotic prescribing decreased resulting in a small net decrease of 5·0% since the campaign’s initiation.

Finally, a community campaign in 2018 which enrolled 21 clinics across ten locations in the USA also targeted HCPs through educational sessions and a cross-clinic comparison of prescribing data, with educational materials also provided to patients [25]. Assessment of the number of antibiotic prescriptions written for unresponsive RTIs showed a 46·0% reduction in prescribing post-intervention (OR: 0·54, 95% CI: 0·42 − 0·66, *p* = 0·001) after controlling for seasonality. However, this level of effect only occurred in two of the eleven clinics included in the study and no significant long-term intervention effect was evident.

Overall, a pooled analysis of the effect of European campaigns on DID’s suggest that implementation of a public campaign may reduce antibiotic consumption by 1·3 to 5·6 DID per 1,000 inhabitants [60]. 

### Other outcome measures

Some campaign and intervention evaluations used other outcome measures. For example, campaign recognition and engagement were used by multiple studies evaluating the Antibiotic Guardian campaign. 26·5% of unique visitors to the Antibiotic Guardian campaign website made a pledge, 10·1% more than for a similar pledge-based campaign conducted in Sicily [29], with social media creating the greatest number of unique visitors to the Antibiotic Guardian website (29·0%) [61]. 

### Similarities between campaigns reported to be effective

Evaluation of fourteen campaigns saw a significant improvement in their primary outcome measure, however there was a lack of homogeneity between these campaigns. Targeting a specific infection type (e.g. respiratory tract infection) was a common theme among campaigns that saw significant improvement in their primary outcome measure, these campaigns (*n* = 10) focused messages on the ineffectiveness of antibiotics at treating RTIs. Key messaging included “Antibiotics are not Automatic” [18, 21, 52–54], “Antibiotics are ineffective for common cold, acute bronchitis and flu” [19], “Antibiotics do not help in cold and flu” [22], and “Do Bugs Need Drugs” [24]. 

Mass media was used to disseminate messages in seven campaigns. The use of TV advertising to promote campaign messages was a recurring feature in mass media campaigns which saw a significant improvement in their primary outcome measure and was often cited as the main source of campaign recognition [19, 34, 38, 55]. Finally, some of these mass media campaigns which were reported as being effective also included an element of HCP education or promotion of HCP-patient interaction. This ranged from feedback to GPs on their antibiotic prescribing based on reimbursement data and facilitation of discussions between HCP and patients [19] to academic detailing, individual prescribing feedback and promotion of rapid streptococcal antigen tests [52] to promotional materials sent to all medical doctors and pharmacists [22], and accredited educational courses for physicians and pharmacists [24, 55]. 

A common theme among local and community interventions that saw a significant improvement in their primary outcome measure was an element of HCP education and using HCP-patient interactions to disseminate information. Part of the intervention implemented by Maor et al. included an explanation by physicians to parents for the reason their child has not been prescribed antibiotics during their visit [20]. Furthermore, Shehadeh et al. employed a similar method of disseminating information by employing pharmacists to provide ten-minute education sessions to the public on a one-to-one basis [46]. Finally, Kandeel et al. and Morgan et al. implemented HCP training programmes focusing on appropriate prescribing for RTIs for all clinical specialties, primary care doctors, as well as pharmacists and physicians working within outpatient clinics respectively [25, 48]. These training programmes were supplemented by educational resources targeting both HCPs, patients, and the public. Finally, the two remaining campaigns used school curriculum and social media combined with site-based resources to share campaign messaging regarding AMR and antibiotic use.

Campaign duration varied from 4 months [46] to 19-years [19] and did not seem predictive of significant improvements in the campaign evaluations primary outcome measure with only mass media campaigns repeated annually.

## Discussion

### Main findings

This review found some evidence suggesting national, community and local campaigns had a significant impact on the studies’ primary outcome measure. Knowledge and attitudes was the most used outcome measure to determine campaign effectiveness, followed by change in antibiotic prescribing rates. Most successful campaigns utilised mass media or HCP interaction with the public or patients to disseminate information. This suggests HCP interaction may be an important source of information or may mediate the pathway from knowledge acquisition to behaviour change in patients. However, further research is needed to gain a better understanding of the effect of HCP interaction on patient behaviours. Heterogeneity between studies made comparing the various public AMR campaigns and interventions challenging. It also created difficulties when drawing conclusions regarding the campaign components which are likely to contribute to a campaign’s success.

Only seven studies included in this review used a robust study design [23, 25, 32, 33, 36, 49, 56]. Five studies used a quasi-experimental or experimental study design combined with non-self-report outcome measures [23, 25, 32, 33, 36] which would enable the effectiveness of the campaign or intervention to be determined with a moderate level of validity and reliability [64]. Furthermore, while measurable outcomes, such as antibiotic prescribing and consumption, are informative due to their effect on AMR [65], few studies reported this in the context of GP consultation and RTI rates. Reporting of antibiotic prescribing rates in context provides information on whether the campaign has effectively raised awareness in the public or whether it has altered actions by HCP to ensure prescribing is appropriate. For campaigns with messaging focused on the self-limiting nature of RTIs, a reduction in consultations may suggest that public knowledge regarding infection prevention and the ineffectiveness of antibiotics at treating viral infections has improved. Antibiotic prescribing was reported in the context of GP consultations in studies evaluating the effectiveness of the 2001 French mass media campaign. Dunais et al. showed a significant reduction in prescribing despite no change in average weekly cases of RTI and bronchitis in under 5-year-olds reported by GPs. This indicates a reduction in prescribing for RTIs suggesting the addition of a local intervention aimed at GPs was more influential than the national mass media campaign aimed at improving public knowledge alone [18]. Chahwakilian et al. also found an initial 10·8% reduction in the proportion of consultations leading to a prescription. However, after four-years, the proportion of consultations resulting in an antibiotic prescription increased by 2·0%, suggesting latter declines in prescribing rates were mainly attributable to a continued decline in consultation rates [52]. This suggests campaign messaging encouraging self-care for RTI within the public was effective and long lasting, while aspects of the campaign aimed at inappropriate prescribing among GPs were less so. However, financial disincentives discouraging GP home visits for minor illness were introduced one-year into the campaign, in 2002, and is therefore likely to also have influenced consultation rates. Furthermore, comparisons made between studies evaluating the French national campaign should be interpreted with caution due to the differing data sources used to report on prescribing and consultation rates.

Findings from observational studies should be interpreted with caution due to potential confounding factors, such as other campaigns running concurrently, which limits our ability to assign impact to one specific campaign. Despite difficulties created when evaluating campaign effectiveness, multifaceted campaigns allow for multiple audiences to be targeted as was occurring in the UK between 2017 and 2019 where the Keep Antibiotics Working, Antibiotic Guardian and eBug campaigns and other HCP focused interventions [66, 67] were running simultaneously with significant improvements in AMR knowledge and awareness being reported by studies evaluating these campaigns.

Most studies only assessed the short-term impact of a campaign or intervention. Two exceptions are the evaluation of the long running mass media campaigns in France and Belgium. These campaigns both highlighted large initial reductions in antibiotic prescribing rates (34 and 10% respectively) following primary campaign waves which have subsequently increased, resulting in much smaller net reductions of 12·8% in Belgium and 12·6% in France [19, 53]. 

Previous research on general mass media health promotion campaigns concluded that short and long-term campaigns have similar levels of effectiveness [68]. This may be due to campaign fatigue resulting from long-term campaigns, causing inattention to campaign messaging, and therefore negatively impacting long-term behaviour change [69]. Therefore, further research is needed, especially in the area of AMR, to determine how to prevent campaign fatigue in the public, especially as health messaging currently saturates daily communication channels [70]. One such way to combat this is for campaigns to require active engagement, for example through making a pledge or interactive teaching sessions, rather than continuing to use static messaging to increase awareness as solely promoting awareness can be ineffective [35, 71]. The majority of included campaigns utilised predominately passive resources, such as TV advertising, posters, leaflets, and lectures from HCPs, with only four campaigns promoting interaction and engagement through pledge-based campaigns or interactive teaching sessions with children [29, 40, 61, 72]. However, those campaigns which have promoted engagement have only assessed change in AMR knowledge or self-reported behaviour which, while measuring the impact of the campaign more directly, makes it difficult to determine whether campaign engagement has led to actual behaviour change.

### Future research

This review has highlighted several avenues for future research, primarily the need for a more standardised and robust approach to evaluating public health campaign effectiveness. The evaluation process needs to be considered and embedded within the initial design and development of the campaign with pre-determined outcome measures collected prior to and following the campaign; therefore, a campaign evaluation framework may be a beneficial tool for campaign developers to utilise. Some guidelines have been developed [73, 74] with one example being the ACME (audience–channel–message–evaluation) framework suggested by S. Noar (2012) which outlines key considerations regarding the audience, channel, messaging, and evaluation used when creating a public health campaign [75]. However, its real-world applicability may be limited due to changes in public health communication since its publication. One method which would allow standardised data to be collected on knowledge of AMR and appropriate antibiotic usage as well as antibiotic use behaviours is the implementation of national surveys which are conducted at regular intervals with examples being the tracking survey which is conducted in England and the Eurobarometer survey [76, 77]. As well as allowing for comparisons between countries, capturing this broader data on public knowledge of AMR and appropriate antibiotic usage would also allow differences in knowledge and behaviours between population groups to be determined, thus allowing campaign messaging or dissemination methods to be more targeted towards specific groups.

Furthermore, very few studies were identified which assessed campaign cost-effectiveness [19, 23, 36, 55], meaning it cannot be determined whether public health campaigns targeting AMR provide a good return on investment in terms of reduced cost of antibiotic prescriptions, reduced burden on HCPs or improved patient quality of life. Future research may wish to establish appropriate methods for conducting a cost-effectiveness analysis on AMR public health campaigns. Including these methods at the early stages of campaign design and development would add to the body of evidence assessing whether public health campaigns impact AMR. Building the body of evidence relating to the cost effectiveness of AMR public health campaigns is also likely to effect policy in this area as it may influence the availability of funding for future campaigns.

Finally, there have been a limited number of publications about campaigns conducted in recent years with only three campaigns launched in the last 5-years [25, 29, 49] and two ongoing campaigns [42, 61]. Methods of communication have changed substantially over recent years with the use of social media becoming increasingly prominent and it is now often cited as a preferred source of healthcare information for many [78]. However, this review highlighted only one study which piloted a social media focused campaign [31]. Furthermore, the majority of research on AMR messaging uses social media platforms which are no longer used extensively, such as Twitter [79], especially by younger populations who tend to have poorer knowledge of AMR and appropriate antibiotic usage [76, 80]. Social media and social media influencers have been shown to promote positive attitudes towards other health topics such as cervical screening [81]. As a result, future research may wish to explore the effectiveness of social media channels, such as Instagram, TikTok, Facebook and WhatsApp, as well as the use of influencers to disseminate information relating to AMR and appropriate antimicrobial usage, and to understand the volume of information and misinformation related to AMR which is available online.

### Strengths and limitations

This review is the first to provide an overview of public health campaigns which aim to improve public knowledge of AMR and appropriate antibiotic use without restricting by research design. A strength of the study is the volume of papers screened, highlighting the broad range of campaigns which have been conducted globally. It also outlines some practical considerations that organisations can implement within their campaigns, namely, to ensure messaging is specific to a certain infection site, such as respiratory tract infections, that HCP education is considered alongside public education and that a mix of media sources are used to disseminate information. However, the review does have some limitations, mainly linked to the use of a rapid review methodology. Firstly, due to the rapid systematic review methodology, a limited number of databases were searched, however reference lists of previously conducted systematic reviews were reviewed and relevant papers extracted in an attempt to mitigate this. Also, the reference software was used to screen for articles containing key words relevant to review with only a subset of papers manually screened and a limited number of articles were screened by a second reviewer (4%). Finally, a quality assessment of included papers was not conducted, however this is not considered essential for rapid reviews, especially when scoping the available literature [82, 83], although any potential methodological issues have been highlighted within the narrative synthesis.

## Conclusions

This review provides some evidence that evaluations of both large and small-scale campaigns reported significant improvements in outcome measures relating to AMR and antibiotic usage. Despite a lack of homogeneity between study designs some common themes emerged between effective campaigns, such as the use of mass media and HCP interaction to disseminate information, as well as the targeting of messaging towards a specific infection site, especially RTI.

However, the frequent use of observational study designs impacts the validity of their findings and makes it difficult to establish causation between the campaign and changes seen in the studies’ outcome measures. Furthermore, the lack of recently conducted campaigns creates difficulties when generalising findings to the public due to changes seen in health literacy following the COVID-19 pandemic. Finally, future work needs to identify a more standardised and robust approach to evaluating public health campaign effectiveness.

### Electronic supplementary material

Below is the link to the electronic supplementary material.

**Supplementary Material 1:** PRISMA 2020 item checklist. Supplementary Material 2. Search Strategies

**Supplementary Material 2:** Search Strategies

**Supplementary Material 3:** PRISMA diagram outlining the study selection process

## Data Availability

The datasets used during the current study are available from the corresponding author on reasonable request.

## Abbreviations

AMR: Antimicrobial resistance
CI 95%: Confidence interval
DID: Defined daily dose per 100,000 inhabitants
EAAD: European Antimicrobial Awareness Day
EU: Europe
GP: General practitioner
HCP: Healthcare professional
OR: Odds ratio
RTI: Respiratory tract infections
UK: United Kingdom
