# Supplementary material for: Assessment of global antimicrobial resistance campaigns conducted to improve public awareness and antimicrobial use behaviours: a rapid systematic review
Source: BMC Public Health. 2024 Feb 6;24:396. doi: 10.1186/s12889-024-17766-w (PMC10848528; doi:10.1186/s12889-024-17766-w)
Supplement: Supplementary file 2 — Supplementary Material 2: Search Strategies [file 12889_2024_17766_MOESM2_ESM.docx]

Supplementary Material 2. Search Strategies

|  | **Ovid ALL Medline** |
| --- | --- |
| 1 | (antibiotic* or anti-biotic* or antimicrobial* or anti-microbial* or antibacterial* or anti-bacterial* or antiinfection* or anti-infection*).tw,kf. |
| 2 | bacterial resistan*.tw,kf. |
| 3 | (antiviral resistan* or anti-viral resistan*).tw,kf. |
| 4 | drug resistan*.tw,kw. |
| 5 | (multiresistan* or multi-resistan*).tw,kf. |
| 6 | AMR.tw,kf. |
| 7 | drug resistance, microbial/ or exp drug resistance, bacterial/ |
| 8 | Anti-Bacterial Agents/ |
| 9 | Anti-Infective Agents/ |
| 10 | Antimicrobial Stewardship/ |
| 11 | 1 or 2 or 3 or 4 or 5 or 6 or 7 or 8 or 9 or 10 |
| 12 | ((public or communit* or population or neighbor?rhood* or educat* or nation* or region* or district* or state*) adj3 (campaign* or intervention* or program* or initiative)).tw,kf. |
| 13 | (communicat* or disseminat*).tw,kf. |
| 14 | (campaign* or mass media).tw,kf. |
| 15 | (message* or engag* or outreach).tw,kf. |
| 16 | social marketing.tw,kf. |
| 17 | (television or tv).tw,kf. |
| 18 | (radio or broadcast*).tw,kf. |
| 19 | (internet or online).tw,kf. |
| 20 | digital.tw,kf. |
| 21 | (social media or facebook or twitter).tw,kf. |
| 22 | (telephone* or cell* phone* or cell?phone* or mobile phone* or smartphone* or smart-phone* or app or apps).tw,kf. |
| 23 | (text messag* or sms).tw,kf. |
| 24 | (email or e-mail or electronic mail).tw,kf. |
| 25 | publicity.tw,kf. |
| 26 | Internet/ |
| 27 | exp Telecommunications/ |
| 28 | Social Media/ |
| 29 | Social Marketing/ |
| 30 | Mass Media/ |
| 31 | Health Communication/ |
| 32 | Health Education/ |
| 33 | Persuasive Communication/ |
| 34 | 12 or 13 or 14 or 15 or 16 or 17 or 18 or 19 or 20 or 21 or 22 or 23 or 24 or 25 or 26 or 27 or 28 or 29 or 30 or 31 or 32 or 33 |
| 35 | ((public or population or community or citizen* or household* or resident* or consumer* or carer* or caregiver* or people or adult* or men or women or mother* or father* or parent* or student* or pupil* or children or adolescent* or teenager*) adj4 (attitude* or belief* or view* or opinion* or expectation* or knowledge or awareness or risk or perception* or behav* or engagement)).tw,kf. |
| 36 | (behav* adj3 (chang* or alter* or modification* or modify* or modifies or modified or intention*)).tw,kf. |
| 37 | cost effectiv*.tw,kf. |
| 38 | ((reduction or reduce* or decrease*) adj4 ("use" or "using")).tw,kf. |
| 39 | (return on investment or ROI).tw,kf. |
| 40 | "outcome measure*".tw,kf. |
| 41 | Awareness/ |
| 42 | Health Knowledge, Attitudes, Practice/ |
| 43 | Health Promotion/ |
| 44 | exp Attitude to Health/ |
| 45 | Motivation/ |
| 46 | Intention/ |
| 47 | Behavior Therapy/ |
| 48 | Public Opinion/ |
| 49 | Program Evaluation/ |
| 50 | Cost-Benefit Analysis/ |
| 51 | 35 or 36 or 37 or 38 or 39 or 40 or 41 or 42 or 43 or 44 or 45 or 46 or 47 or 48 or 49 or 50 |
| 52 | 11 and 34 and 51 |
| 53 | limit 52 to yr="2010 -Current" |
| 54 | exp Animals/ |
| 55 | exp Humans/ |
| 56 | 54 not 55 |
| 57 | 53 not 56 |
