# Supplementary material for: Assessment of global antimicrobial resistance campaigns conducted to improve public awareness and antimicrobial use behaviours: a rapid systematic review
Source: BMC Public Health. 2024 Feb 6;24:396. doi: 10.1186/s12889-024-17766-w (PMC10848528; doi:10.1186/s12889-024-17766-w)

Supplementary Material 3. PRISMA diagram outlining the study selection process, f*rom:*  Page MJ, McKenzie JE, Bossuyt PM, Boutron I, Hoffmann TC, Mulrow CD, et al. The PRISMA 2020 statement: an updated guideline for reporting systematic reviews. BMJ 2021;372:n71. doi: 10.1136/bmj.n71. For more information, visit: <http://www.prisma-statement.org/>


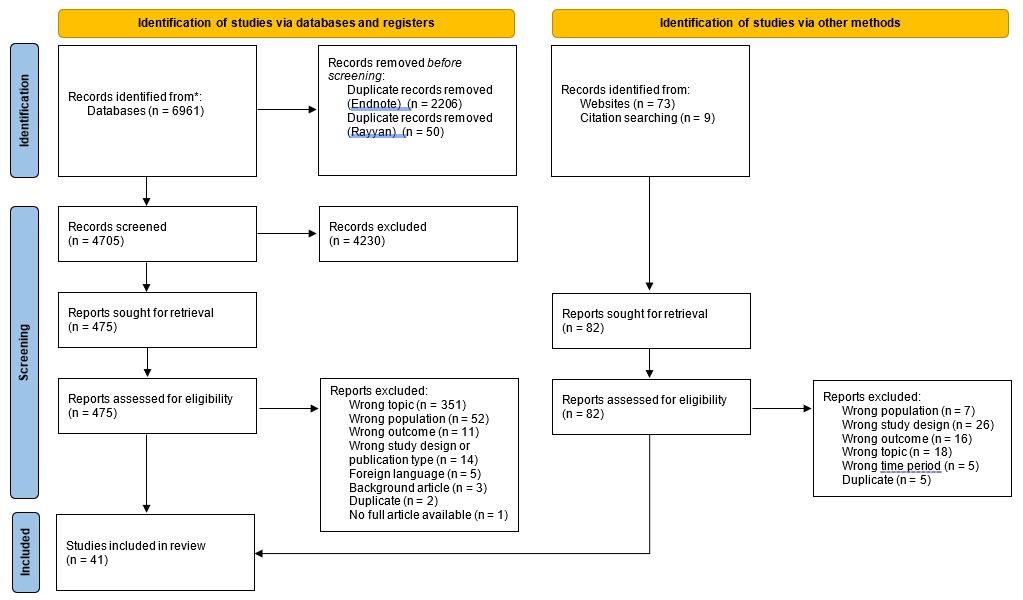

Supplement: Supplementary file 3 — Supplementary Material 3: PRISMA diagram outlining the study selection process [file 12889_2024_17766_MOESM3_ESM.docx]
